# Lectures Illustrative of Various Subjects in Pathology and Surgery

**Published:** 1846-07

**Authors:** 


### MEDICO-CHIRURGICAL

### REVIEW.

JULY, 1846.

### Lectures Illustrative of various Subjects in Pathology and Surgery. By Sir *Benjamin C. Brodie*, Bart. F R.S. Serjeant-Surgeon to the Queen; Surgeon to H. R. H. Prince Albert; Foreign Correspondent of the Institute of France. Octavo, pp. 411. London: Longmans, 1846.

It has often been noticed with regret that the eminent members of our profession who are engaged in extensive practice too frequently neglect the great opportunities they possess of enriching the annals of medical science and practice. This cannot be said of the distinguished author of the volume before us, who shows a due sense of the responsibilities of his position by occasionally recording, for the benefit of his brethren, the results of a large experience, and all must admit that his writings fully justify the great reputation which he enjoys, both in the profession and with the public. To the School of St. George's Hospital, which Sir Benjamin Brodie has always acknowledged himself indebted to for the opportunities of cultivating Pathology and Surgery, he has certainly never been wanting, and since he ceased to give a complete and systematic course of surgical lectures to the students of that institution, he has annually addressed to them a limited number of lectures on the same subjects, which have been continued since he resigned the office of surgeon to the hospital. We learn from the Preface that the present volume contains several of these discourses, which have already appeared in the weekly medical journals, but have undergone various corrections and received such additions as the author's later experience and more mature reflection have enabled him to furnish. Writings of the character of these Lectures are well adapted as a medium for the development of the opinions of an experienced observer, and they are more agreeable to read, and perhaps more profitable to practitioners, than discourses of a more systematic description. The novelty and interest of these Lectures are however much diminished by their previous publication. It sometimes happens that the writings of an author of no note when published in a weekly periodical, though displaying talent, are at the time little read and are soon forgotten, and their disinterment after the writer has acquired some celebrity may be desirable to establish his reputation. But the productions of Sir Benjamin Brodie are little likely to be thus neglected. We know that many of the principles of surgical practice inculcated in the work before us have been served up in

Druit's Vade Mecum, and quoted and taught in every medical school of the metropolis. We may instance the excellent lectures on Varicose Veins and Ulcers of the Legs, on Corns and Bunions, and on Mortification, which we will venture to say have already been more read in the journals than many works of greater pretension. We do not, however, make this remark as an objection to their publication in a collected form, for on the contrary, their popularity is the best proof that, in a corrected and enlarged form, they cannot fail to be acceptable to the profession. Besides these discourses originally addressed to the Students of St. George's Hospital, Sir Benjamin Brodie has also included two lectures, forming part of a course which he delivered as Professor of Anatomy and Surgery to the Royal College of Surgeons of England more than twenty years ago, and which had not been previously published.

The two first lectures are introductory addresses to the students of the Medical School of St. George's Hospital. The first was delivered in 1838, and the second in 1843. These discourses are admirable. They convey in language terse, clear and precise, valuable advice, seasonable hints and useful directions—in fact, just such hints and just such directions as are wanted by the majority of young men on commencing their educational career. They place before the medical student the proper objects and true ends of his professional education, and whilst representing the real difficulties of his course, they offer every encouragement and every incentive which should stimulate a virtuous and honourable ambition. Sir B. Brodie knew well the rock on which the fortunes of many men of ardent minds are shipwrecked, when penning the following passage.

"Let it always be borne in mind that this last (whatever relates to Medical and Surgical treatment) is the real object which you have in view. I address you as future medical practitioners. If, taking another course, you choose to study anatomy and physiology, merely as interesting branches of human knowledge, you are at liberty to do so, and you will be as well rewarded for your labours as if you had applied yourselves to geology, optics, or astronomy. In like manner, if any one apply himself, as a philosopher, altogether to the study of pathology, he will find much in it that may interest himself, and that may be useful afterwards to those who carry their researches further. But as medical practitioners, you must not stop at either one or the other of these points; and, never losing sight of the ultimate object of all your investigations, you must estimate the value of whatever other knowledge you acquire by the degree in which you find it to be directly or indirectly applicable to the healing art." P. 4.

There is not a school in London which cannot furnish examples of poor victims of science, who, little heeding this friendly warning, have blasted their prospects and disappointed the hopes of their friends by acquiring habits of study and tastes for pursuits which have thoroughly unfitted them for the business of life as practitioners.

The second of these two addresses, on the duties and conduct of medical students and practitioners, is far superior to the first. When every page, indeed every sentence, in this lecture contains some judicious counsel or valuable suggestion we feel at a loss to select passages to convey to our readers an adequate idea of its merits. We may quote the following observations on the disadvantages resulting from the neglect of education in early life.

"One business of education is to impart knowledge; but another, and still more important one, is to train the intellectual faculties. To acquire the habit of fixing the attention on the object before you; of observing for yourselves; of thinking and reasoning accurately; of distinguishing at once that which is important from that which is trivial; all this must be accomplished in the early part of life, or it will not be accomplished at all. Nor is the same remark less applicable to qualities of another order; integrity and generosity of character; the disposition to sympathize with others; the power of commanding your own temper; of resisting your selfish instincts; and that self-respect, so important in every profession, but especially so in our own profession, which would prevent you from doing in secret what you would not do before all the world; these things are rarely acquired, except by those who have been careful to scrutinize and regulate their own conduct in the very outset of their career." P. 31.

How encouraging, yet how just, are the author's reflections on the differences in the intellectual powers of competitors in the race of life.

"With respect, however, to the last-mentioned subject, I have no doubt that the difference is not so great as you, or the world generally, may suppose it to be. There are few persons who have not some talent, which, if properly cultivated, may be turned to good account, and he who is deficient in one kind of talent may excel in another. But the greatest talents may be wasted. They may be blighted by indolence; they may be used for base or improper purposes; or they may be directed to too great a variety of objects. It is well indeed for you to have some diversity of study, so as to keep all your mental faculties in wholesome exercise; so that you may not be without some sympathies with those around you, and that you may avoid the evils of narrow-mindedness and prejudice; still, whoever would be really useful in the world, and be distinguished in it, must act to a great extent on the principle of concentration, keeping one object especially in view, and making his other pursuits subservient to it. And let no one sit down in despair and say, 'I have not the abilities of my neighbours, and it is needless for me to exert myself in competition with them.' If you would know what your own powers are you must try to use them. Industry is necessary to their development; and the faculties of the mind, like those of the body, go on improving by cultivation. It is impossible for you to form a right estimate of yourselves in early life, nor can you be rightly estimated by others. The self-sufficient, who do not keep before their eyes an ideal standard of perfection, who compare themselves only with those who are below them, will have an advantage with inexperienced and superficial observers; but I must say that I have never known any one to do any real good in the world, or obtain ultimately a bright reputation for himself, who did not begin life with a certain portion of humility. The greatest men are humble. Humility leads to the highest distinction, for it leads to self-improvement. It is the only foundation of a just self-confidence. Study your own characters; endeavour to learn, and to supply your own deficiencies; never assume to yourselves qualities which you do not possess; combine all this with energy and activity, and you cannot predicate of yourselves, nor can others predicate of you, at what point you may arrive at last." P. 35.

Let the slave to the opinions of others, as well as the hunter after posthumous fame, remember—

"There is no profession in which it is more essential that those engaged in it should cultivate the talent of observing, thinking, and reasoning for themselves, than it is in ours. The best part of every man's knowledge is that which he has acquired for himself, and which he can only to a limited extent communicate to others. You will spend your lives in endeavouring to add to your stores of information; you will, from day to day, obtain a clearer and deeper insight into the phenomena of disease; you will die at last, and three-fourths of your knowledge will die with you; and then others will run the same course." P. 39.

The young practitioner is told

"He who has not a full sense of the responsibilities which it involves, is unfit for our profession; and the anxieties of a professional life are but a wholesome stimulus to diligence and exertion. I say this, supposing them to be kept within reasonable bounds. You may allow your thoughts to dwell on subjects of anxiety until an entirely opposite effect is produced, and life is rendered miserable, and the mind enervated. Such a morbid sensibility is as mischievous on the one hand, as a want of just sensibility is on the other. You must be careful to train the mind so that it may not fall into either of these extremes. Make every exertion to obtain knowledge, and to use it properly; and then keep it in your recollection that there are bounds to human knowledge, and to human powers: and that, in the exercise of our art, we cannot do all that is required of us; for, if we could, pain and misery would be banished from the world, man would be immortal, and the order of the universe would be disturbed. Do not begin life with expecting too much of it. No one can avoid his share of its anxieties and difficulties." P. 43.

We cannot deny ourselves the pleasure of giving one more passage, the spirit and tendency of which exhibit the good sense and good feeling of the writer.

"There are some employments which bring those who are engaged in th in contact more especially with the bad qualities of mankind, their pride, th arrogance, their selfishness, their want of principle. It is not so with your profession. All varieties of character will be thrown open to your view; but nevertheless, you will see on the whole the better side of human nature; much indeed of its weakness, much of its failings, much of what is wrong, but more of what is good, in it. Communicating, as you will probably do, with persons of all conditions, you will be led to estimate others according to their intrinsic qualities, and not according to those circumstances which are external to themselves. You will learn, that of the various classes of which society is composed, no one is pre-eminently good or pre-eminently bad; and that the difference is merely this, that the vices and virtues of one class are not exactly the vices and virtues of another. You will have little sympathy with those prejudices which separate different classes from each other; which cause the poor to look with suspicion on the rich, and the rich to look down upon the poor; and while you cannot fail to perceive the great advantages which education gives, you will acknowledge, that, to be well educated, is not the necessary result of having the opportunity of education; that a bad education is worse than none at all; and that what is called the uneducated classes present many examples, not only of the highest religious and moral principles, but of superior intellect, and of minds stored with valuable knowledge." P. 52.

We need scarcely add that we strongly recommend the perusal of these addresses, especially of the second, to all medical students and young practitioners.

The third Lecture is on the Effects of Strangulation. Sir B. Brodie states—

"The mode of death from strangulation or hanging is sufficiently obvious. 1. The trachea is obstructed, so that air cannot enter the lungs. 2. The blood passing through the lungs does not undergo that change which respiration produces, and which is necessary to life. 3. Dark-coloured blood, which has not been purified by exposure to air, is transmitted to the left side of the heart, and from thence to the brain and other organs. 4. The heart continues to act, circulating dark-coloured blood, but its actions gradually become weaker, and, in the course of a very few minutes, cease altogether." P. 61.

The author's observations on the immediate cause of the cessation of the heart's action, and on the length of time the heart continues to act so as to circulate blood, after respiration has ceased, are full of interest. The description given of the symptoms which arise in the different stages of strangulation, though founded chiefly on experiments made on the lower animals, is no doubt a faithful representation of the phenomena occurring in the human body. Under the head of treatment to be had recourse to in cases of strangulation, excellent directions are given for making artificial respiration, and as there are many occasions besides those of strangulation in which practitioners may be unexpectedly called upon to conduct this process, and as erroneous notions prevail as to the best means of doing it, we may briefly notice Sir B. Brodie's views on the subject. We believe him to be quite right in stating that the only effectual method of supplying the want of natural respiration is that of inflating the lungs, by a pair of bellows, or a syringe, or some similar contrivance. The operator is repeatedly cautioned against the danger of too forcible distension of the air-cells and the impulsion of air into the blood-vessels, through the thin tunics of the capillary vessels of the lungs. It is stated, " under ordinary circumstances, there are three inspirations for one pulsation of the heart and arteries." This is an error, of course accidental, which is too obvious to mislead. Sir B. Brodie no doubt intended to write *there is one inspiration to three pulsations of the heart and arteries.* In cases of obstruction from inflammation and swelling of the mucous membrane of the pharynx and larynx, the lungs can be inflated only by means of a tube introduced into the trachea, and the simplest mode of proceeding is to make an opening with a double-edged scalpel in the space between the cricoid and thyroid cartilages, this situation being more convenient (where time is valuable) than one lower down, on account of the smaller quantity of dissection which is required for the exposure of the trachea in the former case, and the greater liability to hæmorrhage in the latter. We are glad to find that our author does not recommend the curved trochar and canula, which is now sold by most instrument-makers, and is used by some of the surgeons to the Borough Hospitals. We are convinced that this is a very dangerous instrument, its use being attended with considerable risk of wounding the posterior part of the larynx or trachea, and of making an opening into the œsophagus. In cases of strangulation it is not necessary to introduce a tube into the trachea. A tube introduced into one nostril will answer every purpose, and it may be passed without delay. " A short ivory tube, of the size of the anterior aperture of the nostril, and with the projecting rim, to prevent it slipping in beyond your reach, is very convenient for the purpose; but this may not be at hand; and you will find a large elastic gum catheter; a piece of card rolled into a cylinder; or the nozzle of a pair of bellows, to answer the purpose well enough, and at all events any one of these may be used until you are provided with a better apparatus." But bellows may not be at hand, the surgeon must then be content, in the first instance, to inflate the lungs by his breath, or by that of another person, with the aid of a gum tube, or any other tube that can be procured. The upper part of the patient's person should be exposed, and the operator will then know, by observing a gentle elevation of the chest and abdomen, that the lungs are sufficiently inflated. It is needless to give directions as to the exact admeasurement of the air. However good this may be in theory it is practically impossible, A portion of the air escapes by the other nostril and the mouth, and these apertures must not be closed, as they form a safety-valve which will do more towards preventing the over-distension of the lungs than the most ingenious apparatus. The operator must bear in mind that the quantity of air which is required at each artificial inspiration is not only not greater, but that it may even be less, than in natural breathing. An assistant should press the thyroid and cricoid cartilages against the vertebræ, so as to close the upper extremity of the œsophagus, otherwise a portion of air will, at each closure of the bellows, find its way into the stomach, gradually distending it until it prevents the descent of the diaphragm and the entrance of the air into the lungs altogether. Voltaic electricity, which has been recommended in these cases, is shown to be quite inapplicable in surgical practice. A pair of bellows may be obtained anywhere; but a voltaic battery is not to be had at the moment, and requires time for being brought into operation. Sir B. Brodie remarks:—

"It cannot be too strongly impressed on your minds that in these cases for the most part there is no time to be lost. If the natural efforts to respire have actually ceased, the cessation of the heart's action will take place in the course of a very few minutes. If the patient recover from the first effects of the strangulation, but lies with stertorous respiration, and other symptoms of apoplexy, he may cease to breathe altogether at any moment: *and if that action of the heart by which the circulation is maintained should cease, as a consequence of the suspension of respiration, it can never be restored. This I positively assert, after having made it the subject of a very careful investigation.* If others have held a different opinion, it is because they have confounded those feeble and irregular contractions of the heart, which may last for a long time, but which mean nothing, with those regular and powerful movements which are necessary to propel the blood through the system. The most probable means of restoring the action of the heart would seem to be the application of voltaic electricity. But Bichat distinctly states that it has no influence whatever over the involuntary muscles; and without venturing either absolutely to confirm, or absolutely to deny this assertion, I am bound to say, after having made many experiments on the subject, that when the mode of death is that to which the name of asphyxia has been commonly but (according to its etymology) most absurdly and improperly applied, the application of electricity in any form to the heart is altogether useless." P. 82.

Sir B. Brodie is not favorable to venesection in the treatment of strangulation, and is also of opinion that as much benefit may be obtained from keeping the patient in an atmosphere of a moderately warm temperature as from placing him in a warm bath.

In regard to the mode of death from drowning, which is treated of in the fourth Lecture, it is stated to be similar to death the consequence of strangulation; and the want of the due oxygenation or decarbonization of the blood is the sole cause of the animal's destruction. The following is a good account of the phenomena of drowning, as observed on immersing a small animal in water.

"There is first a deep expiration, by which bubbles of air are expelled from the lungs. There is then an effort to inspire; but the effort is ineffectual, there being no air which can be received into the lungs; and a spasm of the muscles seems to prevent the admission of water in any considerable quantity into the trachea. The attempts to breathe are repeated several times; and after each attempt a small quantity of air is expelled from the mouth and nostrils, until the air-cells of the lungs are almost completely emptied. Then the animal becomes insensible, and convulsive actions of the muscles mark the instant when the brain begins to suffer from the influx of the dark-coloured blood. After these convulsions the animal is motionless, and gives no signs of life; but if the hand be applied to the thorax the pulsation of the heart, gradually becoming fainter and fainter, indicates that some remains of vitality still linger in the system. Before the circulation ceases altogether, the muscles of respiration resume their action, and some ineffectual efforts are again made to breathe. It is a remarkable circumstance that the diaphragm continues to exert itself nearly as long as the heart itself, so that the interval between the cessation of the attempts to breathe and the cessation of the motions of the heart, short as it is in animals that die of strangulation, is shorter still in those that perish from drowning. These phenomena follow each other in rapid succession, and the whole scene is closed, and the living animal is converted into a lifeless corpse, in the brief space of a few minutes." P. 86.

Sir B. Brodie believes that the heart is very rarely, if ever, capable of maintaining the circulation for a longer period than five minutes after complete submersion. After remarking that a spasm of the muscles of the glottis seems to prevent the admission of air into the windpipe, he notices some experiments made on animals, and which prove that a very small quantity of water obtains admittance, and states that there is no manifest reason why this should be very injurious.

In his experiments Sir B. Brodie found the action of the heart to be generally more feeble in animals which are drowned than in those which are strangled; and he knows not to what this difference can be attributed, unless it be the additional shock which the former species of death occasions to the nervous system, in consequence of the immersion of the animal in a cold medium which rapidly carries off the animal heat. He agrees with all sound physiologists in totally disbelieving the tales related by travellers of divers who have been capable of remaining under water for twenty minutes, or even for a longer period. Dr. Davy, who resided for a considerable time in Ceylon, found on enquiry that the average time of diving is less than a minute. Similar errors prevail respecting the time that persons have been restored to life after submersion. We are informed in a note that Mr. Woolley, the surgical attendant at the Receiving House of the Royal Humane Society in Hyde Park, believes that very few lives are preserved after four minutes of complete submersion. Two instances, however, of persons who have recovered after having been five minutes under water, have come under his observation.

As the mode of death from drowning is the same as from strangulation, so there must be a great similarity of treatment. We find, however, no suggestions on this subject but what are well known to the profession. Sir B. Brodie confesses, however, "on the whole it must be acknowledged that in both orders of cases the resources of art are limited, and that of those who recover from a state approaching to dissolution a greater number will owe their recovery to unassisted nature, than to the most judicious treatment."

With reference to the mode in which death is produced by a stroke of lightning, Sir B. Brodie remarks, that the facts which he has been able to collect relating to this subject, lead him to the conclusion, that the influence of lightning, or of a powerful shock of electricity, in the majority of cases, is expended chiefly in disturbing, or destroying, the functions of the brain.

It has given us pleasure to call attention to these two lectures, delivered at the Royal College of Surgeons, because Sir B. Brodie's later labours in practical surgery have somewhat eclipsed his former, but scarcely less important, labours in the field of physiology.

In Lecture V, a short account is given of some cases of cysts containing watery fluid, apparently connected with the liver. Two cases are related of fluctuating tumours occurring on the right side, and lifting up the ribs. They were tapped and a watery fluid evacuated. In each instance the wound closed by the first intention, and there was no return of the fluid. It is supposed that these cysts were connected with the liver, and that they were analogous to the cysts occasionally developed in the neck, and also in the testes and spermatic cord in encysted hydrocele. A third case of the kind, in which after puncture the cyst suppurated, and burst into the colon, is also detailed.

In Lecture VI., which treats of ununited fractures, Sir B. Brodie describes the process of union of a fractured bone, but makes no addition to what has long been known on this subject. He next considers the circumstances under which fractures do not unite, noticing most of the causes usually described by surgical writers as preventing a bony union. He remarks—

" In most instances, I cannot doubt that the want of union is to be traced to a peculiar state of the constitution. A gentleman was growing fat, and not liking to do so, he placed himself on a very spare diet, though accustomed to good living previously. After six months of starvation, he broke his arm, and the bone would not unite. I saw him many months afterwards, and there was scarcely any union, even by soft substance. Another patient about whom I was consulted, a lady, also was growing corpulent, and she also thought that she might prevent it, by pursuing a similar system of diet. Some months afterwards she broke her fore-arm, and union did not take place. A young man had been for many months living very low on account of a complaint under which he laboured, and under these circumstances broke both bones of his fore-arm. At the end of several months there was no union. Cases of ununited fracture are not very common, yet here are three among those which have fallen under my observation, in which the want of union seems clearly to be traced to the bad state of the constitution, produced by abstinence from food." P. 125.

He thinks that, in some cases, too tight bandaging by interfering with the supply of blood to the limb, may prevent the union of a fracture. This is very probable. In some experiments on rabbits and other animals he found, after fracturing the femur, and then tying the femoral artery, that until the end of the seventh day there was no commencement of the process of union. But after this period union goes on as usual, owing, it is supposed, to the anastomosing branches having become sufficiently dilated to make up for the obliteration of the femoral artery. Mr. Travers, however, in his work on Constitutional Irritation, has recorded a case of fractured femur, accompanied with injury to the popliteal artery, in which, although the femoral artery was tied, the fracture was soundly united in six weeks. Sir B. Brodie does not notice the effects of one end of the fractured bone being cut off from its supply of blood from its nutrient artery, as interfering with the union in some cases, a circumstance pointed out, if we recollect rightly, in a paper published a few years back in the Transactions of the Medico-Chirurgical Society.

Sir B. Brodie reviews the various means which have been employed for the purpose of procuring an union of the fracture. He gives no sanction to the operation of cutting down on the broken ends of the bones, and sawing off a portion of each of them, and supposes that no modern surgeon, having a moderate share of prudence, would undertake it. In reference to the introduction of the seton in these cases, he remarks that the result of the practice in this country appears to be, that sometimes it has succeeded in the upper extremities, but that where it has been performed on the lower extremities, as far as he knows, it has only succeeded in a single instance. The operation is uncertain, and the result tedious. Sir B. Brodie speaks favourably of the treatment by pressure proposed by Mr. Amesbury, and states that it succeeded perfectly in three cases which were attended with him. The success, in one of these at least, was not so complete as is represented, since it appears that there was so much yielding motion between the upper and lower portions of the fractured femur, "that it was plain that the union could be merely ligamentous." In this mode of treatment the pressure must be considerable, so as to cause some inconvenience to the patient, both from pain and from swelling of the limb below. But the inconvenience is only temporary.

"The principle of Mr. Amesbury's practice is simply that of keeping the ends of the bones in perfect repose, and at the same time applying pressure, particularly on the broken surfaces, so as to keep them in the closest possible contact with each other. Of course no general rule can be laid down as to the mode of attaining this object. In a case of transverse fracture, one kind of apparatus must be employed, in one of oblique fracture another, and in one of comminuted fracture a third. The apparatus will also differ accordingly as it is a fracture of the arm, the fore-arm, the leg, or the thigh. In a case of oblique fracture a very simple apparatus will do all that is required. Secure the limb by fastening it to a single rather broad wooden splint. Apply a pad of thick leather on each side of the fracture, and then a tourniquet, by which the two opposite surfaces of bone may be kept firmly squeezed against each other. By means of the tourniquet the pressure may be easily regulated, and increased or diminished as the patient can bear it. The best kind of tourniquet is not the common one, known under the name of Petit's, but one, which occupies a smaller space, invented by the late Mr. Savigny, and sold by Philp and Whicker in St. James's Street.

"I do not say, however, that this method always succeeds. I had tried it in the case of the little boy whose case I have already mentioned, (on whose leg I afterwards used the seton,) and without advantage. There was another patient in this hospital on whom it was tried for a considerable time under Mr. Amesbury's observation, and no union was effected: and it appears that Mr. Amesbury has met with some cases in his own private practice, in which he has adopted it, and no doubt done ample justice to it, but in which it has failed. Still it has proved a very successful method on the whole, and certainly very much more successful than any other." P. 135.

The subject of Lecture VII. is sero-cystic tumours of the female breast. This is the most complete account of the disease that we have met with. This affection was improperly termed by Sir A. Cooper "hydatid disease of the breast," its pathological character being quite distinct from true hydatid formations, which occasionally occur in the female mamma. The first perceptible indication of the disease is a globular tumour imbedded in the glandular structure of the breast, and to a certain extent moveable underneath the skin. Sometimes there is only one such tumour; at other times there are two or three, or many more. In most instances the disease is confined to one breast. There seems to be little doubt that the cysts are originally formed by a dilatation of the lactiferous tubes. The diagnosis is generally easy, but occasionally the tumour is so deeply seated, that even a very experienced person may not at once recognise its nature in the first instance, and may be led to suppose that it is a medullary tumour or a chronic abscess, or any thing else, rather than what it really is. It occurs at the middle period of life, and is more common in single than in married women. The pathological history of the disease is thus described.

"*First:* A greater or less number of membranous cysts are generated in the breast, containing serum. The latter is at first of a light yellow colour, and transparent, but afterwards becomes of a darker colour, and opaque. There is reason to believe that these cysts are formed by a dilatation of portions of some of the lactiferous tubes.

"*Secondly:* Morbid growths or excrescences are generated from the inner surface of one or more of these cysts, projecting into their cavities. These excrescences seem to consist of albumen or fibrine, which, after some time (if not immediately), becomes organized. They are covered by a thin delicate membrane, which is reflected over them from the inner surface of the cyst; but whether they are originally formed between two layers of the membrane of the cyst, or whether they are at first mere deposits of fibrine or albumen on the inner surface of the cyst, a thin membrane being formed on their surface afterwards, remains to be determined by future observations.

"*Thirdly:* There is some reason for believing that a similar growth of fibrinous substance may take place from the external surface of the cysts, connecting different cysts with each other; but this point also may, perhaps, require to be illustrated by further investigations.

"*Fourthly:* Under certain circumstances the cysts become completely filled up by the morbid growths, so that their cavities are obliterated, the tumor being thus converted into a solid mass, in which, however, the remains of the cysts are perceptible; and this is the prelude to a still further change, in which the greater part of the cysts have wholly disappeared, a solid mass of an indistinctly laminated texture occupying their place.

"*Fifthly:* If one of the membranous cysts be artificially laid open, or if it burst from over-distension with serum, the fibrinous excrescence, from its inner surface being no longer restrained by the pressure of the skin, increases in size, and protrudes externally in the form of a fungus, giving to the tumour a new and more formidable character.

"In this last stage of the disease, it is evident that spreading ulceration, sloughing, and hæmorrhage, the usual results of an ulcer occurring in a diseased structure, must ensue, for which our art furnishes no other means of cure than the removal of the affected parts by a surgical operation." P. 149.

In the early stage of this disease, the treatment recommended, is the application of a stimulating embrocation to the skin. In the majority of cases thus treated, the tumour or tumours have entirely disappeared; in others, without disappearing altogether, they have become very much reduced in size; and, in only a few instances, in which the treatment was not very rigidly pursued, it was productive of no manifest advantage. The application generally employed is the following.

"℞. *spiritûs camphorati, spiritûs tenuioris,* āā. ℥iijss; *liquoris plumbi diacetatis,* ℥j.; *fiat embrocatio.*

"I have directed the patient to soak a piece of flannel, once folded, in this embrocation, and to apply it so as to cover that part of the breast in which the tumor is situated, renewing the application six or eight times in the day and night, until the skin becomes inflamed; then to omit the application for two or three days, but to resume the use of it as soon as the inflammation has subsided. The period of time during which it is necessary to pursue this method of treatment varies in different cases. In some, all that can be desired is accomplished in the course of three or four weeks; in others, it must be continued, with occasional intermissions, for some months. Other stimulating applications may be occasionally substituted for that which I have just mentioned. Several blisters may be applied in succession; each of them being kept open for a few days with the savine cerate; or a solution of ʒj. of iodine in ℥j. of alcohol may be applied to the skin once or twice daily, by means of a large camel's hair brush. On the whole, however, I am led to believe that the embrocation is more efficient than any thing else." P. 153.

But this simple treatment is wholly inefficient after the growth of solid substance has commenced. In this more advanced period of the disease, no good is to be expected, except from the removal of the entire breast; and such an operation may be had recourse to with every prospect of success, the disease being entirely local. Sir B. Brodie states that it undouhtedly is not malignant. It may go on to inflammation and ulceration, and the ulcer may spread and slough and bleed, but it does not contaminate the constitution. He adds, however, "still, I am not prepared to say that it may not, under certain circumstances, and in peculiar constitutions, assume a malignant character; this being no more than may happen to almost any morbid growth." That it may in an advanced stage acquire a true carcinomatous character we have no doubt whatever; but we believe that this is only an occasional, and not a common, result of the disease.

In Lectures VIII. and IX. Sir B. Brodie treats of Varicose Veins and Ulcers of the Legs, the formation and pathological causes of which, he describes with his usual accuracy. We find nothing, however, but what is well known to practical surgeons. In speaking of the application of plaster in these cases he recommends the soap plaster spread on the *fungus* or *amadon* used for the lighting of cigars. Being to a certain degree elastic, it admits of being applied in a single piece and makes a very uniform pressure. This plaster is sold by Mr. Weatherfield, in Henrietta-street, Covent-garden. Sir B. Brodie states that, in private practice, he frequently recommends a bandage which is made of stocking web, which, we quite agree with him, is very convenient, being easily applied, and making a very equal pressure : indeed, for comfort and efficiency, we believe this bandage to be far preferable to the best made elastic stockings. He adds, that it cannot well be used by the poorer classes of society, being more expensive in the first instance, and being also good for nothing after it has been washed a few times. This is a mistake. A coarser kind of bandage well adapted for poor persons may be purchased for a shilling, and it will last six months, and bear washing many times. Sir B. Brodie gives no countenance to operations on the vena saphena, and mentions cases in which, after division of, or after tying this vein, the patients have died from venous inflammation. He remarks—

"There are indeed no circumstances here to justify the performance of a dangerous operation. You may perform such operations to get rid of a disease still more dangerous, but you have no right to perform an operation attended with such a degree of danger as can be appreciated, in order to get rid of a disease which is not dangerous; and no one can say that varicose veins belong to the class of dangerous diseases. But still there is another reason against having recourse to this operation. I do not believe, from what I have formerly seen, that it permanently benefits the patients. It is true that they appeared to go out of the hospital much relieved; but where I had the opportunity of seeing them one or two years afterwards, I always found them as bad as ever. Indeed I am by no means certain that the benefit which the patients appeared to derive, in the first instance, was the result of the operation; and I am more inclined to believe that it arose from their having been necessarily kept for some time in bed in the horizontal posture. Patients with varicose veins always seem to improve under these circumstances. But I may observe further, that there appears to be no reason why, in ordinary cases of varicose veins, the obliteration of the *saphena* should do any good, and that there are better grounds for believing that it will do harm. I have already explained to you that pressure on large venous trunks causes an obstruction of the blood in passing through them, and that this is one common cause of varices." P. 186.

Lecture X. contains some interesting observations "on the Cases of Scirrhous Tumours of the Breast which require an Operation." Sir B. Brodie notices the difference of opinion that has existed amongst surgeons of eminence as to the propriety of operating in scirrhous disease of the mamma, and after insisting on the importance of removal of the entire mamma in cases in which an operation is considered desirable, he remarks, "you may divide scirrhous tumours of the breast into two classes: one, where there is a conversion of the gland of the breast itself into the scirrhous structure, there being no well-defined margin to it; the other, where there is a scirrhous tumour imbedded in what appears to be otherwise a healthy breast, as if it were altogether a new growth, there being a well-defined boundary to it." In the first order of cases, the operation not only never succeeds in making a permanent cure, but it rather hastens the progress of the disease. The patient dies within two or three years, and probably much sooner, from an effusion of fluid into the cavity of the pleura. In another order of cases, where the skin is contaminated, there is no chance of the operation making an ultimate or permanent cure. The retraction of the nipple and tucking in of the skin are also regarded as unfavourable symptoms, and forming objections to the operation. The presence of indurated glands in the axilla, the adhesion of the scirrhous tumour to the pectoral muscle or ribs, indications of malignant disease in other organs, are all circumstances sufficient to forbid an operation with a view to an ultimate cure. Having taken away these cases, there are very few left in which it is right to propose an operation as affording a chance of permanent cure. An operation is proper in cases free from the objections here mentioned. It may be performed, too, with a still better prospect of success in cases of scirrhous tumour unconnected with the breast though attached to it, and also in cases of scirrhous tumour of the nipple. Our author remarks that there may be circumstances which justify the surgeon in performing the operation for the removal of a scirrhous tumour of the breast, not in the expectation of a permanent cure, but with the view to afford the patient a respite and relief from present suffering, and he mentions several cases in which life appeared to have been prolonged by an operation.

"Of course you are here called upon to exercise no small degree of discrimination; and especially you should reject altogether those cases in which the skin is distinctly contaminated by the disease, whether it be that there are scirrhous tubercles in it, or that it be converted into the brawny structure which I have formerly described. In neither of these cases will the patient obtain even a respite by submitting to an operation." P. 206.

Sir B. Brodie observes that the operation is not free from danger, and admits that he has lost patients after it. He adds, however, "But where the breast is small; where the patient is otherwise healthy, and not much advanced in life; and where you do not starve the patient either before the operation or after it; and are also careful that there shall be as little loss of blood as possible;—there the danger of the operation is comparatively trifling."

Lecture XI. is on Corns and Bunions, a subject which Sir B. Brodie has not disdained to treat of—and though these affections have been described by fashionable chiropodists in handsome quarto works, we believe that the brief observations of Her Majesty's Serjeant-Surgeon contains the best description of them, and the best directions for their relief and cure which have hitherto been published. Those of our readers who may suffer from corns or bunions, or who may wish for information as to their causes and treatment, cannot do better than read this lecture.

The subject considered in Lecture XII. is one of considerable interest—the administration of mercury in cases of syphilis. We are glad to perceive that Sir B. Brodie rejects the notion of treating this disease without mercury, and of mercury aggravating the disease instead of curing it. Experience proves to him that we have hitherto found no remedy having the same power of extinguishing the venereal poison as mercury. In answer to the objection that it may do great harm, he justly remarks, "In this there is nothing at all remarkable, for (with the exception, perhaps, of sarsaparilla,) I do not know any medicine capable of doing great good, that may not, under certain circumstances, operate as a poison. I saw a gentleman very nearly killed by an over-dose of quinine: others have died in consequence of the imprudent exhibition of the iodide of potassium; and others have been killed by arsenic. A remedy that is strong enough to do good is almost invariably strong enough to do harm, if it be not used properly." He states that mercury is not to be given as a matter of course in all cases of syphilis ; but the general rule is, that it should be given. We think this remark might have been qualified by the addition of "in the primary and secondary stages of the disease," for, in tertiary syphilis, we quite agree with Ricord, that mercury is generally inapplicable. Sir B. Brodie next describes several conditions in which it would not be right to give this remedy. In reference to the best mode of administering it, he, in common with most good practical surgeons, gives the preference to inunction in cases where the symptoms of syphilis are not of the very mildest character. He seems to think that we have rather gone back than advanced in the treatment of this disease, and that the mercurial treatment as employed by the late Mr. Pearson during the greater part of his life, was as nearly perfect as possible, and that it was much more successful than the less careful treatment of modern practitioners. There is certainly much force in these remarks. If mercury be necessary, it should be given so to affect the system as to eradicate the disease, or it will do more harm than good. If the constitution cannot bear this action of the remedy, then do not give it at all. No advantage is gained in attempting to get rid of the disease or to keep the symptoms under by small doses of mercury. They become more confirmed and more difficult of cure when the disease is treated in this way, and there are other remedies which are more efficacious as palliatives, and at the same time do less injury to the system than mercury. Our author's observations on the treatment of syphilis in infants are deserving of attention.

"Children are sometimes born with syphilis, the father or mother having been affected with it. The child looks thin, and is of small size; and, instead of thriving, becomes thinner and thinner. At the end of three weeks it is covered by a red scaly eruption; there are aphthæ in the mouth, with chaps about the lips and the anus. The symptoms are well marked, and tell you at once the nature of the disease. I have tried various ways of treating such cases. I have given the grey powder internally to the child, or some kind of mercury to the wet nurse. But the mercury given to the infant by the mouth gripes and purges severely; that given to the wet nurse cannot be depended on; and at all events the latter is a very cruel and scarcely justifiable practice. The mode in which I have treated such cases for some years past is this: I have provided a flannel roller, on one end of which I have spread some mercurial ointment,—say a drachm, or more; and I have applied the roller, thus prepared, not very tight, round the knee; repeating the application daily. The motions of the child produce the necessary friction: and the cuticle being thin the mercury easily enters the system. This causes neither griping nor purging; in a child it does not even in general cause soreness of the gums; but it cures the disease. Very few of those children ultimately recover in whom the mercury has been given internally; but I have not seen a single case in which this other method of treatment has failed." P. 245.

As mercurial fumigations have been recommended by a modern writer on Syphilis, our readers will like to know the opinion of this method of treatment entertained by Sir B. Brodie. He states, "I have used the mercurial vapour-bath with success in several cases where it was my object at once to affect the system ; but I have found that Mr. Pearson's objection to it is very well founded; namely, that it is difficult in this way to regulate the mercurial action. You may affect the system too much, or too little; and you may be taken unawares by the patient's gums becoming all at once excessively sore." In the review of Mr. Parker's work on Syphilis, in our preceding number (p. 456), we expressed a similar opinion.

The following remarks, with which this Lecture is concluded, are so true and practical that we cannot forbear quoting them.

"I have spoken of the necessity of administering mercury, not only till the symptoms are relieved, but for a considerable time afterwards. But you may ask, whether a long course of mercury be not more likely to injure the constitution than a short one? Undoubtedly it is; and that is the very reason why you should prefer a long course. If the course be a short one, the disease is sure to return; you have then to repeat it, and again the disease reappears. Thus you have repeated courses; and not only is the system weakened by the mercury, but the disease, whenever it does return, assumes a more formidable character than before. But if, on the other hand, you put the patient through a long course in the first instance, such a frequent recurrence to the use of mercury will be unnecessary. A patient who takes mercury for a chancre, for a month or five weeks, may probably never want it again; but if he take it only for a fortnight, he has secondary symptoms, and then it will be required for at least six weeks, perhaps for ten; so that that which is a short course at first becomes a long one in the end." P. 249.

Tic Douloureux, or facial neuralgia, forms the subject of Lecture XIII. The symptoms are briefly but graphically described. It is worthy of notice that the disease affects only one side of the face. Sir B. Brodie has never met with a case in which it affected both sides. Diseased teeth have been supposed to give rise to this painful affection, but he never knew a case in which a patient was relieved of a genuine *tic douloureux* by the extraction of a tooth. Cases in which it appeared to depend on diseased bone are mentioned, but these are rare occurrences. "It is a great mistake to suppose that diseased or dead bone is an ordinary cause of this disease." Various other sources to which the disease may be traced are noticed, as hysteria, disorders of the digestive organs and organic disease in the brain. Sir B. Brodie observes:

"There are many cases in which you cannot trace the *tic douloureux* to its real source. There is something or another, somewhere or another, in the system, which acts as a source of irritation to the nerves of the face; but where that something is, and what it is, we cannot discover. Indeed, generally speaking, I should say that to trace local nervous affections to their origin is one of the greatest difficulties that we meet with in the practice of our art. The disease may be in one part of the body, and the pain or spasm which it produces may be in another. I have known a patient have neuralgia of the foot, which depended on a stricture of the urethra, and which, whenever it occurred, was invariably relieved by the use of a bougie. I have known another patient have neuralgia of the foot depending on internal piles, which came on when the piles were protruded through the anus, and went away when they were reduced. I have known a spasmodic wry neck, or a nervous pain in the back, to alternate with insanity." P. 259.

He adds that, there is good reason to believe that the seat of those nervous communications on which such sympathies depend is for the most part not in the nerves themselves, but in a higher place—in the brain, or in the spinal cord.

In the treatment of this painful affection, Sir B. Brodie strongly objects to operations for dividing the trunks of the nerves; observing, that the irritating cause, whatever it may be, manifestly acts not on the extremity of the nerve, but on its origin; and both reason and experience prove that the division of the nerves below the origin is of no service. It is altogether an unscientific operation. After making some excellent practical observations on the treatment of neuralgia in cases where the disease may be referred to some obvious causes, and in those in which the disease cannot be traced to its real source, the question is asked—

"But having tried all ordinary means without benefit, are you to go on *ad infinitum* tormenting the patient with medicine? The first rule of our art is to do no harm, and if you have had recourse to all reasonable expedients without benefit, it is not advisable for you to go on making further experiments. *No one can be dosed constantly with medicine without the health being ultimately injured by it; and if you have not some reasonable grounds for giving medicine, you have no right to run the risk of doing harm by its continued exhibition.* It is much more wise, as well as much more honest, when you do not know what to do, to do nothing, and to advise your patient to wait and take the chance of the pain subsiding of itself, as in fact it does in a great many instances." P. 265.

Sir B. Brodie has no great faith in the veratrine ointment; and the extravagant manner in which this application has been lauded as a remedy for neuralgia, leads him to conclude this lecture with the following seasonable observations.

"The statement of its effects which had been published promised a great deal too much, and I should have expected more if it had promised less. I shall take this opportunity of observing, that I am not disposed to try indiscriminately all the new remedies which in these days are being constantly brought before society; nor can I think well of this modern fashion of resorting on all occasions to novel methods of treatment. I advise you, if you wish to succeed in your profession, and to be useful to society, to pursue a different course. Make yourselves masters of the old remedies. Learn how to handle them, and what good they will do, and, as a general rule, have recourse to them in the first instance. If the old remedies fail, and you are at a loss as to what you should do, then, and not till then, have recourse to the new ones. If you always begin with new remedies you throw away the valuable results, not only of your own experience, but of the experience of those who have gone before you. You have to begin, as it were, *de novo;* and the first consequence of this will be that you will not cure your patients; and the second, that you will have none to cure. I should be very sorry to see the march of science impeded by an unjust apprehension of experiments and innovations; but, surely, there is a broad enough line between a discreet and prudent use of new remedies, and that indiscreet and hasty use of them which we find to prevail at present in the practice of our profession, and especially in that of its junior members." P. 268.

Lecture XIV. consists of practical observations on fatty or adipose tumours. Mention is made of a kind of fatty tumour which occurs occasionally, but which has not been described by surgical writers.

"In the cases to which I allude the tumour is not well defined; in fact there is no distinct boundary to it, and you cannot say where the natural adipose structure ends and the morbid growth begins. I will relate to you the history of one of several cases of this kind that I have met with, and this will explain as much as I know of the matter. A man came to this hospital several years ago having a very grotesque appearance; there being an enormous double chin

(as it is called) hanging nearly down to the sternum, and an immense swelling also on the back of his neck formed by two large masses, one behind each ear, as large as an orange, and connected by a smaller mass between them. He said that the enlargement had begun to show itself three or four years before, and had been increasing ever since. They gave him no pain; nevertheless they made him miserable, and in fact had ruined him. The poor fellow was by occupation a gentleman's servant, and having so strange an appearance no one would take him into his service. I gave him half a drachm of the *liquor potassæ* three times daily, and gradually increased the dose to a drachm, dissolved in small beer. When he had taken the medicine for about a month the tumours were sensibly diminished in size. He went on taking the alkali, and the tumours continued to decrease. It was just then that iodine began to have a reputation, much indeed beyond experience has proved it to deserve, for the cure of morbid growths, and I left off the *liquor potassæ*, and prescribed the tincture of iodine instead. The effect of this change of treatment was remarkable. The patient lost flesh, while the tumours increased in size. Of course I omitted the iodine, and prescribed the *liquor potassæ* a second time. Altogether he took a very large quantity of the latter medicine, and left the hospital very much improved, with directions that he should continue to take it with occasional intermissions. I had lost sight of him for some time, when it happened that I was requested to visit a patient in Mortimer Street. I did not observe the servant who opened the door, but as I was leaving the house he stopped me, saying that he wished to thank me for what I had done for him. It was this very patient. He was so much improved in appearance that he was enabled to obtain a situation as a footman. There were still some remains of the tumours, but nothing that was very remarkable. I have seen some other cases of the same kind, in which the exhibition of very large doses of the *liquor potassæ* appeared to be of great service. But I have not had the opportunity of trying it, or of knowing the results in every case; and I am informed that in some cases it has been given to a considerable extent without manifest advantage." P. 276.

These tumours feel like fat; but they may be distinguished from common fatty tumours by their having no well-defined boundary, and by their being less soft and elastic. Such deposits may probably take place in any part of the body, but our author has seen them more frequently in the neck than anywhere else. Sir B. Brodie describes another kind of tumours, which also, as far as he knows, are not described by surgical writers. "They are situated in the subcutaneous adeps, give no pain, and are not tender to the touch; they have a well-defined margin, and are of a somewhat firmer consistence than common fatty tumours, to which, in other respects, they bear a great resemblance. They grow to a certain point, then remain stationary, while others show themselves elsewhere; until, at last, there is no part of the trunk or extremities in which they are not to be met with, varying in size from that of a pea to that of a small walnut." If any of these tumours attained a large size they might be removed with the knife, but as they are numerous it would be absurd to think of removing the whole in this manner. In these cases he has given the *liquor potassæ* in large doses with service. In one case, the tumours nearly or quite disappeared under its use. He thinks it probable that, in cases of fatty deposits, the *liquor potassæ* operates in the following manner: the oily part of the tumour combines with the alkali, is taken into the circulation and thus carried off. He gives as much as a drachm and a half, in a large quantity of fresh small beer. It seems to act better in this than when given in any other liquid, and the beer does not disagree with the stomach because the alkali neutralizes every drop of vinegar which it contains. Sir B. Brodie agrees in the opinion of Sir Astley Cooper, that a fatty tumour will sometimes take on the action of a malignant disease, and become a malignant tumour. The case, however, related in confirmation of this opinion, does not seem to us a satisfactory example.

In the next six Lectures the subject of Mortification is very fully treated of. They contain the best account of this process, and of its causes and treatment, that we know of. Our notice of the volume has been so full, that we have only space for a few remarks and extracts of an important practical character, or that may be new to our readers. In reference to the use of ammonia in mortification Sir B. Brodie remarks, "It is a temporary stimulus; but alcohol, prudently administered, is much better; and my observations lead me to suspect that large doses of ammonia, if persevered in for a considerable time, tend to depress the vital powers, and lessen the chances of recovery." Whenever putrid matter is pent up round a slough of the cellular membrane, the system is poisoned by it. The sulphuretted and carburetted hydrogen gas evolved during the decomposition of dead animal matter, seem to pass, in part at least, into the circulation, producing the most dangerous symptoms. The incisions which relieve the tension of the skin allow these noxious gases to escape, and the relief which this affords to the patient is very great. The following is a remarkable example.

"I was called, some few years since, to see a gentleman, who appeared to be actually on the point of death. His extremities were cold; his pulse barely perceptible. It was doubtful whether he was sensible or not. He made, on being roused, several imperfect attempts to speak, but could say nothing intelligible. Below the right hypochondrium there was a considerable tumor, the skin being of a dark red colour, on the verge of mortification. On examination with the fingers, I perceived a sort of emphysematous crackling, and an imperfect fluctuation. Having made a free incision, I discovered, underneath the discoloured skin, what might be called a quagmire of slough. A small quantity of putrid matter escaped; but there escaped also such a quantity of noisome and offensive gas, apparently sulphuretted hydrogen, that I could scarcely bear to remain in the room. The stench pervaded the whole house, and even could be perceived in the garden round it. Within two minutes after the performance of this operation, so trifling in appearance, but so important in reality, the patient looked up, and said, quite distinctly, 'What is that you have done which has made so great a difference in my feelings?' At the same time the pulse returned at the wrist; and from this moment he recovered, without any further unfavourable symptoms. After a few days sloughs came away, probably of muscle, cellular membrane, and peritoneum, in a confused mass, and, with them, a gallstone of moderate size; explaining, to a certain extent, at least, the origin of the disease." P. 299.

In Lecture XVII. there are some valuable observations on the use of the different kinds of Caustics. In bites by rabid dogs, the depth of the wounds being uncertain, Sir B. Brodie observes :—

"The best caustic, I apprehend, for you to use on these occasions is the caustic potash; and for this reason: that it dissolves the parts with which it comes in contact, and that afterwards the dissolved caustic penetrates still further beyond the part to which it has been actually applied. If the tooth penetrate to the cellular membrane, by the time that you are consulted some of the saliva may have reached the cells beyond, and if you apply the nitrate of silver, or the nitric acid, these will coagulate the fluids and harden the solids, while the caustic potash becoming diffused will follow the course of the saliva. A convenient way of applying the caustic on these and on some other occasions is this: melt it in a silver or platina spoon, and, when melted, dip into it the blunt end of a probe. It will come out with a varnish of the caustic upon it; dip it in again until the button of caustic has attained a sufficient size. By means of a probe thus armed you may carry the caustic even into a very narrow wound, so that you are sure it will penetrate wherever the dog's tooth has penetrated; after which, from the particular nature of the caustic (as I have just explained) you may be certain that it will penetrate still further, and as far as the poison can have reached." P. 325.

Caustics, it is remarked, may often be used very advantageously for the purpose of destroying diseased lymphatic glands, forming the base of ill-conditioned ulcers in the groin, which are indisposed to heal. A kind of caustic is required which will lie in the substance of the diseased glands, and destroy their internal structure, as well as their outer surface. The following caustic, used by the late Mr. Pearson, answers this purpose. "It consists of one ounce of crumb of bread, two drachms of the bichloride of mercury, and one drachm of red oxide of lead. These are to be mixed together, kneaded with the fingers, and formed into a sort of paste. The paste should be rolled into little conical troches, and these, if left to dry, become hard, like what are called bread seals. These troches may be stuck into the enlarged gland like pins into a pin-cushion. In the course of a little time they begin to act, as the patient knows from the pain produced. The pain lasts for some hours, and if a sufficient number of the troches be employed, the whole of the gland is at once destroyed. If any portion remains, it is easy to destroy it by repeating the process." In the removal of small nævi and small morbid growths of different kinds, Sir B. Brodie prefers the caustic to the use of the knife. He states, "a wound always heals much more readily after the application of caustic, than after the use of the knife. Take two cases: if you destroy one tumour of a given size by the knife, and the other, supposed to be of the same size, by caustic, in spite of the time occupied by the separation of the slough, the sore in the latter case will be healed sooner than that in the former." It is less formidable to the patient than the knife, and is likewise attended with less risk. For example, an attack of erysipelas seldom follows the use of caustic; certainly much less frequently than after the use of the knife. The application of caustic to tumours on the scalp must be made with great caution, as appears from the following case.

"A surgeon applied the caustic potash to the scalp, with a view to make an issue in a man's head, who was labouring under a head-ache and nothing else. When the slough had separated a piece of the occiput was exposed, as large as half-a-crown, or larger. The patient was soon seized with a set of strange symptoms, and died. It was found that the dura mater had become detached from the inside of the bone, just opposite the part where the pericranium had been destroyed on the outside; and it was clear that the sloughing of the dura mater was the cause of the man's death." P. 337.

When caustics are used, it is prudent to have some counter-agent at hand to stop their action on the sound parts around. "Acids may be neutralized by alkalies; caustic potash may be neutralized by vinegar, or by a solution of the diacetate of lead. If you are afraid of nitrate of silver burning the neighbouring parts, its action may be neutralized by common olive oil. A solution of the bicarbonate of potash will decompose chloride of zinc ; and so with other caustics."

Sir B. Brodie does not agree with Dupuytren, who described the gangrene that occurs in old age as the result of arterial inflammation. He has examined the bodies of a great many old persons who have died with mortification of the toes, and has always found some morbid condition of the arteries of the affected limb. In the very great majority of cases there is extensive ossification of the arteries of the thigh and leg; in many cases they are not only ossified, but some of them are contracted and obliterated. This we believe to be the view entertained by all the best English pathologists, whilst the majority of the French adhere to the erroneous views of Dupuytren. It has been said that mortification of the toes in old persons is often the result of disease in the heart itself. This does not correspond with the results of our author's experience. It is true that he has known persons who had disease in the heart to die of mortification of the toes; but then there was always enough in the condition of the arteries of the limb to account for the mortification independently of the other disease. Sir B. Brodie recommends neither depleting nor a decidedly stimulating treatment, but a nutritious diet and such stimulants, suited to the previous habits of the patient, as do not occasion heat of skin nor raise the pulse. He advises the limb to be wrapped in carded wool, and we are not surprised to find that he strongly objects to amputation even when the mortification is arrested. "If you apply your knife to living parts, you will probably bring on a fresh attack of mortification. Leave the separation altogether to the natural process, which will do all that is required."

Lecture XXI. the last in the volume, is on Chronic Abscess of the Tibia. A case is related of extremely painful enlargement of the lower end of the tibia which led to amputation of the limb. A cavity, as large as a small chesnut and containing pus, was discovered in the diseased bone. This occurred in 1824. It was clear that, if an opening in the tibia had been made with a trephine the limb might have been saved. In a case of painful enlargement of the tibia, in which Sir B. Brodie was consulted about two years after the occurrence of the preceding case, he made an opening into the cancellous structure of the bone with a trephine, and having given vent to some pus, the patient became cured. Four other cases, three of enlargement of the lower end of the tibia, and one of the upper, are related. They were all trephined with a successful result in giving exit to pus and curing the patient of a most painful affection. The operation has also been twice successfully performed by Mr. Liston. The following important questions present themselves. What are the circumstances that would lead the surgeon to suspect the existence of abscess in the tibia? And, supposing it to be probable that such an abscess exists, what are the exact steps of the operation to be performed for its relief?

"When the tibia is enlarged from a deposit of bone externally—when there is excessive pain, such as may be supposed to depend on extreme tension, the pain being aggravated at intervals, and these symptoms continue and become still further aggravated, not yielding to medicines, or other treatment that may be had recourse to, then you may reasonably suspect the existence of abscess in the centre of the bone. You are not to suppose that there is no abscess because the pain is not constant; on the contrary, it very often comes on only at intervals, and in one of the cases which I have related there was, as I then mentioned, an actual intermission of seven or eight months. After the disease has existed a certain number of years, indeed, the pain never entirely subsides, but still it varies, and there are always periods of abatement and of exacerbation. The combination of circumstances which I have described will fully justify you in making an opening into the bone with a trephine. But how will it be if you are mistaken? This will not often occur; but if it should, the taking out a circle of bone can be of no consequence; no injury follows the operation: it is unattended with danger. The operation itself is very simple. You expose the surface of the bone, and make a circular opening with a trephine at that part where there seems to be some tenderness and some pain on pressure. One principal thing to be attended to is that you have a proper trephine. You do not want so large an one as for the cranium, and it must be somewhat differently constructed. Those which lie on the table are made for the purpose. One is of very small diameter, but, generally, it is quite sufficient. The common trephine is made with a projecting rim or shoulder, and if there be much enlargement of the bone, it will not penetrate deep enough to reach the abscess. It is true that you may break away the bone afterwards, by means of a chisel, but the operation may be more easily performed with a trephine having no projection, which will at once penetrate to the abscess, however deep it may be, and render the chisel unnecessary." P. 405.

After the operation, the bone soon granulates, the space is filled up by a sort of fibrous substance, and the wound cicatrizes. Sir B. Brodie states that, if the operation were not performed, the patient may continue in torture for a great number of years, losing all the best part of his life; or a worse event than that may take place. The abscess may lead to disease in a neighbouring joint and in this way prove destructive to life. Cases are given in which abscesses in the head of the tibia had caused serious disease of the knee-joint. These cases " show that it is not safe to leave an abscess in the extremity of the tibia beyond a certain time; that the joint is always in danger, and that the perforation of the bone is the only remedy. Even if you were mistaken in your diagnosis no harm can arise from the operation."

A case is mentioned of a young gentleman who had a painful enlargement of the middle of the humerus. It was trephined freely, but no matter escaped. The bone was very hard and compact, and the trephine was with difficulty made to penetrate through it. The wound healed, the relief was complete, and the patient continued quite well. Sir B. Brodie supposes " that this was a case of chronic inflammation of the humerus, and that taking out the piece of bone from the centre, probably, partly by relieving the tension, and partly by a discharge of matter from the bone unloading the vessels, accounted for the relief which the patient obtained from the operation."

Notwithstanding the result of this case, it cannot be questioned that the greatest care and caution are required in determining the necessity for an operation for the evacuation of a supposed abscess in the substance of a bone. We have been informed that, not long since, a limb was amputated by a hospital surgeon, in consequence of a suspected abscess in the head of the tibia of a painful character, and implicating the knee-joint. On examination of the limb after removal, the joint and tibia were found to be quite healthy, and the complaint proved to be hysterical.

We here terminate our notice of these interesting Lectures. We have not attempted more than to furnish an outline of the subjects treated of, and a condensed account of the more important contents of the volume, giving such extracts as may appear likely to be useful to the majority of our readers. Sir B. Brodie's position in the profession and great experience are such that his practical writings must be considered as beyond the reach of criticism, at least by those of more limited observation. Time will be the best test of the value of the principles inculcated and of the suggestions for the treatment of disease. We strongly recommend our friends not to rest satisfied with this notice, but to read the volume itself, and we can promise them that, by doing so, they will obtain a clearer insight into many obscure subjects of surgical pathology, and become better armed to overcome the difficulties of practice. It now only remains for us to return our most hearty thanks to the author for this valuable contribution to surgical literature, and to express a cordial hope that he may have health and leisure to fulfil the expectations which he has held out of publishing a second Series of his Lectures.

